# In Situ Investigation of the 3D Mechanical Microstructure at Nanoscale: Nano-CT Imaging Method of Local Small Region in Large Scale Sample

**DOI:** 10.1155/2014/806371

**Published:** 2014-02-27

**Authors:** Bo Dong, Feng Xu, Xiao-fang Hu, Hong-yan Qu, Dan Kang, Ti-qiao Xiao

**Affiliations:** ^1^CAS Key Laboratory of Mechanical Behavior and Design of Materials, University of Science and Technology of China, Hefei 230026, China; ^2^Shanghai Institute of Applied Physics, Chinese Academy of Sciences, Shanghai 201204, China

## Abstract

To investigate the local micro-/nanoscale region in a large scale sample, an image reconstruction method for nanometer computed tomography (nano-CT) was proposed in this paper. In the algorithm, wavelets were used to localize the filtered-backprojection (FBP) algorithm because of its space-frequency localization property. After the implementation of the algorithm, two simulation local reconstruction experiments were performed to confirm its effectiveness. Three evaluation criteria were used in the experiments to judge the quality of the reconstructed images. The experimental results showed that the algorithm proposed in this paper performed best because (1) the quality of its results had improved 20%–30% compared to the results of FBP and 10%–30% compared to the results of another wavelet algorithm; (2) the new algorithm was stable under different circumstances. Besides, an actual reconstruction experiment was performed using real projection data that had been collected in a CT experiment. Two-dimensional (2D) and three-dimensional (3D) images of the sample were reconstructed. The microstructure of the sample could be clearly observed in the reconstructed images. Since much attention has been directed towards the nano-CT technique to investigate the microstructure of materials, this new wavelet-based local tomography algorithm could be considered as a meaningful effort.

## 1. Introduction

With the rapid development of material science, advanced experiment and investigation technique have been developed and used to investigate the micro-/nanoscale morphology and mechanical properties of samples. It is well known that the mechanical properties are determined by the material itself and its internal microstructure. It has been found that the mechanical properties of materials changed greatly as their internal structure varies from macroscale to micro-/nanoscale. So it is very important to find the technique that is capable of investigating the micro-/nanoscale structure and mechanical properties. The major investigation techniques included electron microscope and ultrasonic inspection. But considering that the structure to be investigated was extremely tiny and the sample was sometimes placed in external field (including stress, heat and electric), those techniques couldnot complete the investigation under these circumstance. But the CT technique was a good choice because of its nondestructive and noncontacted characteristics. More importantly, 2D and 3D images of the internal microstructure of materials could be obtained by CT and the images could achieve micro-/nanoscale spatial resolution. Taking the nano-CT device in Beijing Synchrotron Radiation Facility as an example, its spatial resolution has reached 30 nm/pixel.

However, the number of pixels is limited by the size of the X-ray acquisition lens. If the spatial resolution was improved, the view field decreased. Once again taking the nano-CT device as an example, there were 1024 pixels along the horizontal direction. So the transverse size of the view field was about 30 um. If the size of a sample exceeded that number, the sample could not be correctly reconstructed using conventional image reconstruction algorithm (i.e., the FBP). On the other hand, fabricating tiny samples was usually a challenge and the samples may be too small to represent the real structure of the material. Thus, how to complete exact tomography under the condition of relative large sample and small view field has become a problem that needs urgent solution. The problem could also be described as local reconstruction in which only the projections of a small local region were acquired and used. The corresponding image method was referred to as local reconstruction algorithm. During the past years, several different local reconstruction algorithms have been proposed by Smith [1–3], Katsevich [4, 5], Noo and Pan [6, 7], and so forth.

Wavelets have received much attention in the past few years. As wavelets are designed to have many good characteristics, it is possible to apply wavelet theory to image reconstruction field. The research on that aspect has been developed rapidly. Some local tomography algorithms have been proposed in recent years [8–12].

In this paper, a new wavelet-based reconstruction algorithm was proposed and implemented. At first, the reason why the FBP could not complete local reconstruction was discussed. Then a new local reconstruction algorithm based on wavelet was proposed and implemented. The key to this algorithm is that many wavelets have the space-frequency localization property. The difference between this new method and another wavelet reconstruction algorithm was also discussed. Next, simulation experiments were performed to test the algorithm. The results of the new algorithm were presented in this paper, together with the results of other algorithms. Besides, the new algorithm was applied to an actual CT experiment and the reconstructed images of local area were also presented.

## 2. Algorithm and Implementation

The most commonly used image reconstruction algorithm is the FBP. However, it is not competent to reconstruct a local region with only local projection data. The reason is that the ideal ramp filtering function in the algorithm is truncated by a spectral window in frequency domain and the windowed ramp filter is unbounded in space domain. So the key point of local reconstruction is to find a window function which ensures that the windowed ramp filter is bounded both in frequency and space domain. A possible alternative is the wavelet due to its time-frequency localization property. So the wavelet-based local reconstruction algorithm was proposed. More details about it will be given below.

At the beginning, the terminology and definitions required in the subsequent discussions will be briefly introduced. In this paper, the following notations are used. The *n* dimensional Euclidean space is denoted by *R* ^*n*^. The Fourier transform in *R* ^*n*^ is defined by $\hat{f}\left(\vec{\omega }\right)=\int_{R^{n}}f\left(\vec{x}\right)e^{i2\pi \vec{\omega }\cdot \vec{x}}d\vec{x}=F\left[f\right]$. The inverse Fourier transform is defined by $f\left(\vec{x}\right)=\int_{R^{n}}\hat{f}\left(\vec{\omega }\right)e^{-i2\pi \vec{x}\cdot \vec{\omega }}d\vec{\omega }=F^{-1}\left[\hat{f}\right]$. The convolution of two measurable square-integral functions *f*(*x*) and *g*(*x*) is denoted by *f*(*x*)∗*g*(*x*), with identical notation for discrete convolution.

### 2.1. The Nonlocality of FBP Coming from the Window Function

The goal of CT is to reconstruct an image from a set of its line-integral projections. In the 2D case, the projection of *f*(*x*, *y*) at an angle *θ* is as follows:

(1) $$\begin{array}{c} R_{\theta }f\left(s\right)=\int_{}^{}\int_{-\infty }^{\infty }f\left(x,y\right)\delta \left(s-x\cos\theta -y\sin\theta \right)dx\;dy. \end{array}$$

Equation (1) is known as the Radon transform of *f*(*x*, *y*).

Conversely, function *f* could be reconstructed from the projection data *R*_*θ*_ *f*(*s*) by

(2) $$\begin{array}{c} f\left(x,y\right)=\int_{0}^{\pi }\int_{-\infty }^{\infty }\left(R_{\theta }\hat{f}\right)\left(\omega \right)e^{j2\pi \omega (x\cos\theta +y\sin\theta )}\left|\omega \right|d\omega \;d\theta . \end{array}$$

The above formula can be implemented in two steps: the filtering step and the backprojection step.

The filtering step can be written as

(3) $$\begin{array}{c} {\hat{Q}}_{\theta }\left(\omega \right)=R_{\theta }\hat{f}\left(\omega \right)\left|\omega \right|. \end{array}$$

The above formula indicates that the Radon transform data is filtered by |*ω*|. This filter is referred to as the ideal ramp filter.

And the backprojection step is

(4) $$\begin{array}{c} f\left(x,y\right)=\int_{0}^{\pi }Q_{\theta }\left(x\cos\theta +y\sin\theta \right)d\theta . \end{array}$$

Because the ideal ramp filter |*ω*| is not bounded in frequency domain, it is expedient in practice to multiply this operator by a spectral window *W*(*ω*) as

(5) $$\begin{array}{c} {\hat{Q}}_{\theta }\left(\omega \right)=R_{\theta }\hat{f}\left(\omega \right)\left|\omega \right|W\left(\omega \right). \end{array}$$

The above operation can be converted to space domain as

(6) Qθ(s)=F−1[Rθf^(ω)|ω|]∗F−1[W(ω)]=H∂Rθf(s)∗F−1[W(ω)],

where *H* is the Hilbert transform and ∂ is ordinary differentiation.

**Figure 1:**
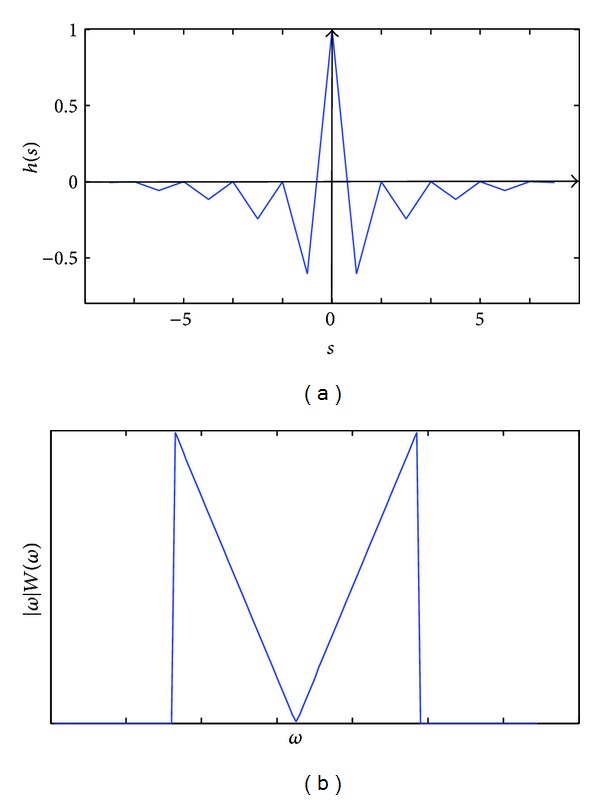
The truncated ramp filter by Hamming window. (a) Space domain. (b) Frequency domain.

Generally, the Hamming window is chosen to be *W*(*ω*). In this case, the windowed ramp filter |*ω*|*W*(*ω*) is bounded in frequency domain, but it is not bounded in space domain (see Figure 1). As a consequence, in order to calculate the convolution in (6) at a point, not only those projections near that point, but also those far away from that point are required. It means that the inverse Radon transform based on (2) cannot be accomplished locally. This property is known as the nonlocality of the FBP algorithm.

Considering the equivalent relation that *F* ^−1^[|*ω*|*W*(*ω*)] = *H*∂*F* ^−1^[*W*(*ω*)], the spreading of the support of the windowed ramp filter in space domain will not occur if the Hilbert transform of a window function is compactly supported. It has been noted that if a function has numbers of vanishing moments, then its Hilbert transform will decay very rapidly at infinity [11, 12]. If a compactly supported function has this property, then the essential support of its Hilbert transform should not be large. This property is fundamental to the wavelet local reconstruction algorithm, which will be discussed below.

### 2.2. Localization of FBP by Using Wavelet as the Window Function

For the reasons outlined above, the functions which are compactly supported and have several vanishing moments are wanted. This second condition will ensure that the functions remain compactly supported after the differential and Hilbert transform process and will ensure that a reconstruction from local data can be accomplished. Wavelets are generally designed with many vanishing moments. So they can be used as the window function in local reconstruction algorithm. In this section, a wavelet-based reconstruction algorithm is presented. The key point of the algorithm is that each projection is filtered by four different wavelet filters, respectively. The filtering formula comes from (6) but the window function is replaced by the Radon transform of 2D wavelet functions:

(7) $$\begin{array}{c} Q_{\theta }^{A}\left(s\right)=H\partial R_{\theta }f*R_{\theta }\left(\Phi \left(x,y\right)\right),\\ Q_{\theta }^{D_{1}}\left(s\right)=H\partial R_{\theta }f*R_{\theta }\left(\Psi_{1}\left(x,y\right)\right),\\ Q_{\theta }^{D_{2}}\left(s\right)=H\partial R_{\theta }f*R_{\theta }\left(\Psi_{2}\left(x,y\right)\right),\\ Q_{\theta }^{D_{3}}\left(s\right)=H\partial R_{\theta }f*R_{\theta }\left(\Psi_{3}\left(x,y\right)\right). \end{array}$$

In (7), the four 2D wavelet functions Φ(*x*, *y*), Ψ_1_(*x*, *y*), Ψ_2_(*x*, *y*), and Ψ_3_(*x*, *y*) are composed by the 1D orthogonal wavelet basis Ψ(*x*) and scaling function Φ(*x*):

(8) $$\begin{array}{c} \Phi \left(x,y\right)=\Phi \left(x\right)\Phi \left(y\right),\\ \Psi_{1}\left(x,y\right)=\Phi \left(x\right)\Psi \left(y\right),\\ \Psi_{2}\left(x,y\right)=\Psi \left(x\right)\Phi \left(y\right),\\ \Psi_{3}\left(x,y\right)=\Psi \left(x\right)\Psi \left(y\right). \end{array}$$

**Figure 2:**
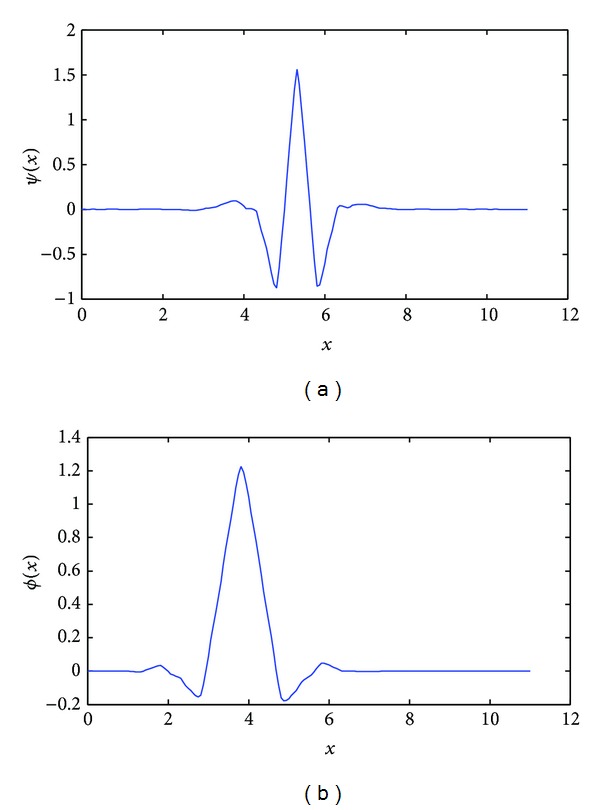
An example of wavelets. (a) Coiflets. (b) Scaling function of Coiflets.

Many kinds of wavelet have been designed by researchers so far. For example, Daubechies, Coiflets, Symlets are commonly used in signal and image processing. Most kinds of wavelet come from a corresponding unique function called the scaling function (see Figure 2). The scaling function has some similar properties with wavelet. It is compactly supported and has high order vanishing moments.

As wavelet and scaling functions are both compactly supported and have many vanishing moments, the functions *H*∂*R*_*θ*_ *g*(*x*, *y*) for each *θ* will decay rapidly outside the support interval, where *g*(*x*, *y*) stands for the four functions denoted in (8). Therefore the filtering in (7) can be accomplished locally. In order to calculate the filtered projection at a point, only a few projections near that point are required. This is the reason why this wavelet-based reconstruction algorithm can complete local tomography.

Besides, the filtering process in (7) can reduce aliasing and high-frequency noise. To explain this it is better to switch (7) into the frequency domain form:

(9) $$\begin{array}{c} {\hat{Q}}_{\theta }\left(\omega \right)=R_{\theta }\hat{f}\left(\omega \right)\left|\omega \right|\hat{g}\left(\omega \cos\theta ,\omega \sin\theta \right), \end{array}$$

where *g* is one of the four functions denoted in (8).

It is obvious that the filter at a certain angle is the multiplication of the ideal ramp filter and the slice of the Fourier transform of the 2D wavelet at the same angle:

(10) $$\begin{array}{c} H_{\theta }^{A}=\left|\omega \right|\hat{\Phi }\left(\omega \cos\theta ,\omega \sin\theta \right)=\left|\omega \right|\hat{\Phi }\left(\omega \cos\theta \right)\hat{\Phi }\left(\omega \sin\theta \right),\\ H_{\theta }^{D_{1}}=\left|\omega \right|{\hat{\Psi }}_{1}\left(\omega \cos\theta ,\omega \sin\theta \right)=\left|\omega \right|\hat{\Phi }\left(\omega \cos\theta \right)\hat{\Psi }\left(\omega \sin\theta \right),\\ H_{\theta }^{D_{2}}=\left|\omega \right|{\hat{\Psi }}_{2}\left(\omega \cos\theta ,\omega \sin\theta \right)=\left|\omega \right|\hat{\Psi }\left(\omega \cos\theta \right)\hat{\Phi }\left(\omega \sin\theta \right),\\ H_{\theta }^{D_{3}}=\left|\omega \right|{\hat{\Psi }}_{3}\left(\omega \cos\theta ,\omega \sin\theta \right)=\left|\omega \right|\hat{\Psi }\left(\omega \cos\theta \right)\hat{\Psi }\left(\omega \sin\theta \right). \end{array}$$

**Figure 3:**
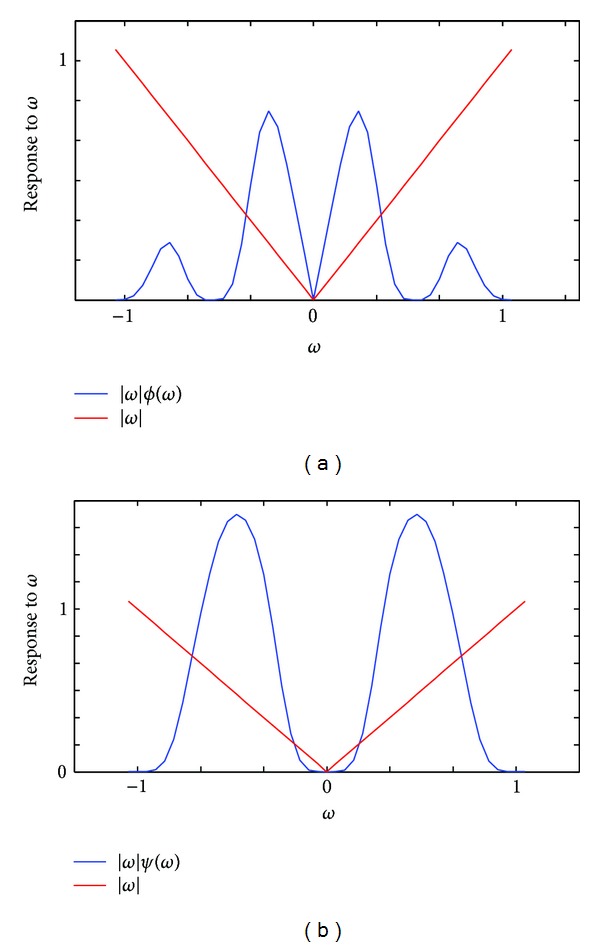
Spectrogram of (a) ramped scaling function and (b) ramped wavelet function. In comparison, the ideal ramp filter was also curved in red line.

These angle-depended filters are simplified into $\left|\omega \right|\hat{\Phi }\left(\omega \right)$ and $\left|\omega \right|\hat{\Psi }\left(\omega \right)$ at special angles like *θ* = 0 or *θ* = (*π*/2). The simplified form can be referred to as the ramped scaling function and the ramped wavelet function.  Figure 3 shows the spectrogram of them. It can be seen that the amplitude of the ramped scaling function and the ramped wavelet function at high frequency is lower than that of the ideal ramp filter. Thus aliasing and high-frequency noise can be reduced after filtering.

### 2.3. Implementation and Comparison with Other Wavelet Algorithm

In the following, the new wavelet-based reconstruction algorithm is summarized in four steps.

- For each projection data at angle *θ*, calculate its differentiation and Hilbert transform.
- The results that have been obtained in step (I) are filtered as the formulas in  (7).
- Filtered projections are back projected using (4) and the results are, respectively, the approximation coefficient, the horizontal detail coefficient, the vertical detail coefficient, and the diagonal detail coefficient.
- The four coefficients are composed into the reconstruction image by 2D inverse wavelet transform.

However, it's worth mentioning that another wavelet algorithm has been proposed in [13]. The main difference is that in [13] the differential operator and the Hilbert operator are conducted to the 2D wavelet function, instead of the projection data. So the filtering step can be written as

(11) $$\begin{array}{c} Q_{\theta }\left(s\right)=R_{\theta }f*H\partial R_{\theta }g\left(x,y\right). \end{array}$$

**Figure 4:**
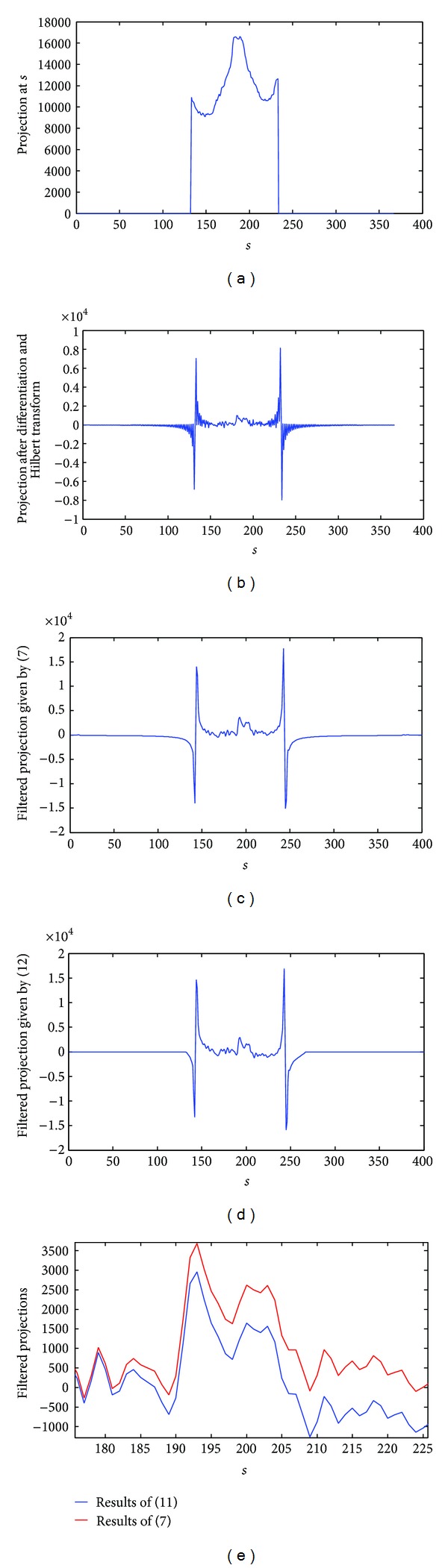
The projection of the Shepp-Logan head phantom at *θ* = 0 was truncated and filtered by different methods. The original length of the projection is 367, but only 100 of them were preserved and the rest were set to zero. In this figure, the horizontal axis represented the index of projection line, the vertical axis represented the projection value before and after filtering. (a) Truncated projection at *θ* = 0. (b) Projection after differentiation and Hilbert transform. (c) Filtered projection given by (7). (d) Filtered projection given by (11). (e) Comparison of the filtered projections. Only a part of projections was shown in this figure.

It can be derived that (7) and (11) are equivalent under continuous situation. But they give different results in practice because actual local projection data are truncated and sampled discretely. Thus the local projection can be considered as a compactly supported function. But generally it does not have vanishing moment. According to (7), after the differentiation and Hilbert transform, the original compactly supported projection will no longer be compactly supported. And after the convolution with the wavelet function, the result is still not bounded (see Figures 4(a), 4(b), and 4(c)). On the other hand, according to (11), after the differentiation and Hilbert transform the wavelet function is still compactly supported. And after the convolution with the truncated projection, the support of the result is almost the same to the projection (see Figure 4(d)). Therefore the two filtering methods provide different filtered projections (see Figure 4(e)). This difference will further lead to a different reconstructed image.

## 3. Experiments and Results

In this section, two different phantom images were introduced to the local reconstruction experiment. The reconstruction algorithms included FBP and wavelet reconstruction algorithm proposed in this paper and in [13]. To evaluate the reconstructed images, three evaluation criteria were used. The experimental results have shown that the filtered-backprojection method is not suitable for local reconstruction; the two wavelet reconstruction algorithms can complete the mission, but the one proposed in this paper performed better than the other one.

### 3.1. Phantom Images and Criteria

**Figure 5:**
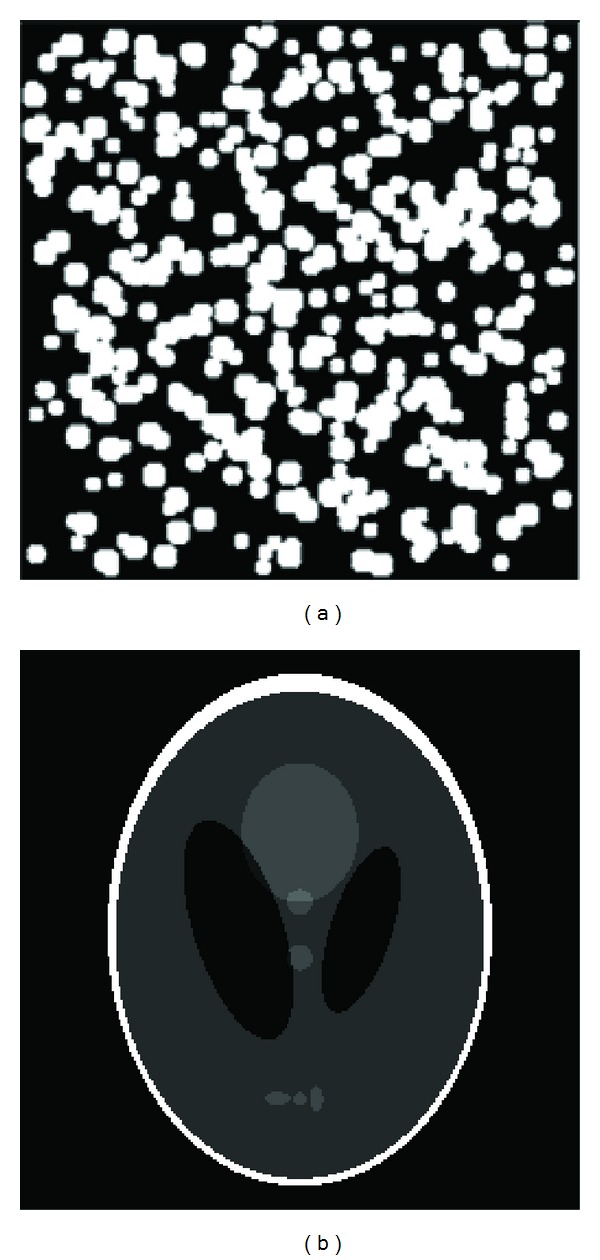
Two phantom images. (a) Stacked particles phantom. (b) Shepp-Logan head phantom.

The model images used in the experiment were the stacked particles phantom and the Shepp-Logan head phantom (see Figure 5). The size of the two images was 256 by 256 pixels and the local area was a disk of 50 pixels in the center of the image. The stacked particles phantom could be considered to contain more high-frequency components because they were composed of a large number of irregularly stacked particle elements whose radius is about 5 pixels. And this image included only two colors: black and white. The Shepp-Logan head phantom was composed of several ellipse elements with different areas and grayscales. In general it could be considered to contain relatively more low-frequency components.

In the following were three criteria to objectively evaluate the differences between original and reconstructed images.

(1)  Normalized mean square criterion *d*:

(12) $$\begin{array}{c} d={\left[\frac{\sum_{u=1}^{N}\sum_{v=1}^{N}{\left(T\left(u,v\right)-R\left(u,v\right)\right)}^{2}}{\sum_{u=1}^{N}\sum_{v=1}^{N}{\left(T\left(u,v\right)-\overset{-}{T}\right)}^{2}}\right]}^{1/2}, \end{array}$$

where *T*(*u*, *v*) and *R*(*u*, *v*) denoted the grayscale of pixels in phantom and reconstructed images. $\overset{-}{T}$ denoted the mean value of the phantom image. *d* = 0 represented exact reconstruction of the phantom. Larger *d* indicated greater error. Besides, *d* was more sensitive to large errors caused by individual pixels.

(2)  Normalized average absolute distance criterion *r*:

(13) $$\begin{array}{c} r=\frac{\sum_{u=1}^{N}\sum_{v=1}^{N}\left|T\left(u,v\right)-R\left(u,v\right)\right|}{\sum_{u=1}^{N}\sum_{v=1}^{N}\left|T\left(u,v\right)\right|}, \end{array}$$

*r* = 0 meant that the reconstructed image was same to the phantom. And like the previous criterion, larger *r* indicated greater error. But *r* was more sensitive to small errors caused by most pixels.

(3)  Standardized covariance criterion *c*:

(14) $$\begin{array}{c} c=\left|\frac{\sum_{u=1}^{N}\sum_{v=1}^{N}\left(T\left(u,v\right)-\overset{-}{T}\right)\left(R\left(u,v\right)-\overset{-}{R}\right)}{\sqrt{\sum_{u=1}^{N}\sum_{v=1}^{N}{\left(T\left(u,v\right)-\overset{-}{T}\right)}^{2}\sum_{u=1}^{N}\sum_{v=1}^{N}{\left(R\left(u,v\right)-\overset{-}{R}\right)}^{2}}}\right|, \end{array}$$

*c* = 0 indicated that the reconstructed image and the phantom image was completely irrelevant. As the degree of correlation between the two images became higher, the value of *c* became closer to 1.

### 3.2. Results and Discussion

**Figure 6:**
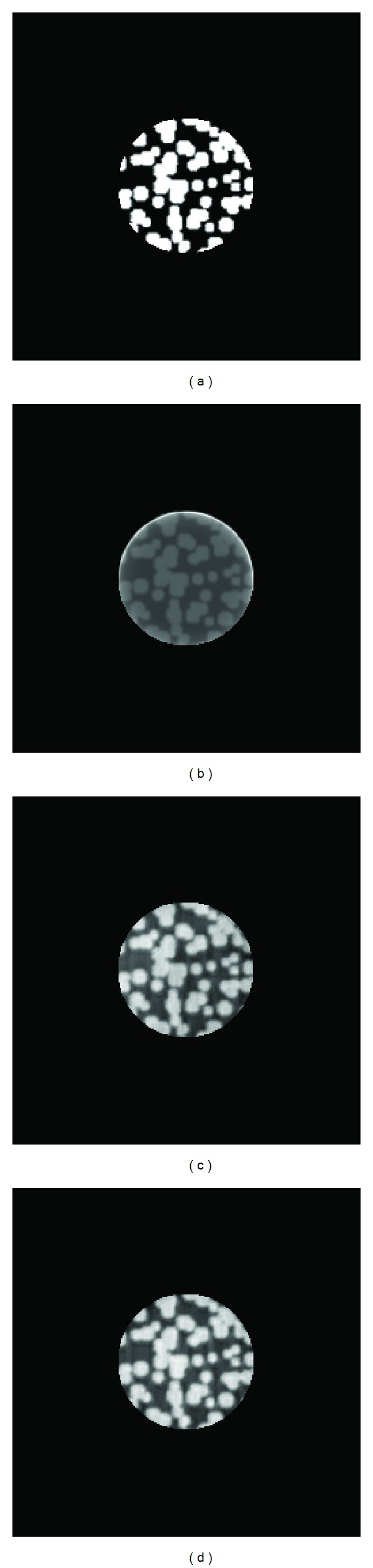
Reconstructed images of the local area in stacked particles phantom. (a) Local area in the phantom. (b) Local reconstruction using FBP. (c) Using wavelet method in [13]. (d) Using new wavelet method in this paper.

**Figure 7:**
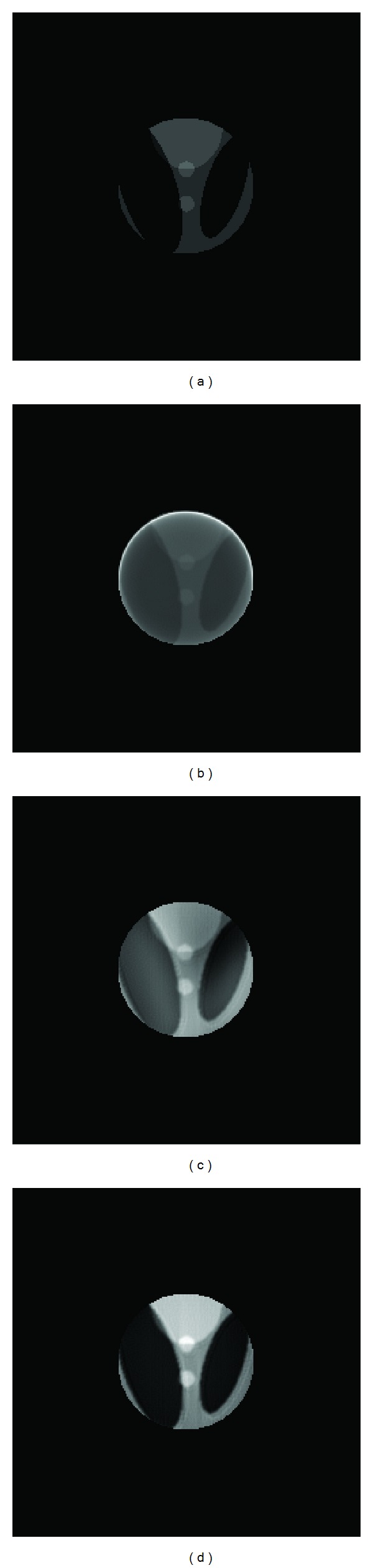
Reconstructed images of the local area in Shepp-Logan head phantom. (a) Local area in the phantom. (b) Local reconstruction using FBP. (c) Using wavelet method in [13]. (d) Using new wavelet method in this paper.

The reconstructed images of the local area in stacked particles phantom were shown in Figure 6 and Table 1. And Figure 7 and Table 2 showed the reconstructed images of the Shepp-Logan head phantom. As a supplement, the relative changes of the parameters compared with the results of FBP were also calculated and listed in the tables.

From the figures and tables, the following could be found.

(1) Among the three algorithms, the FBP did not perform well in local reconstruction. The boundaries in reconstructed images were indistinct and the grayscale of the majority pixels was greatly distorted. The standardized covariance values were less than 0.9 for both phantoms. The algorithms based on wavelets could complete local reconstruction with higher quality than FBP. But in the images reconstructed by the method in [13], there existed a visible difference in grayscale among the pixels inside an element (see Figure 7(c)). At last the new wavelet algorithm performed best generally. Not only the boundaries were clearly reconstructed, but also the average grayscale of each element is close to the origin image.

(2) For the wavelet method in [13], the criteria values changed greatly according to different phantom images. As for the new wavelet method, the relative change of *d* and *r* did not vary much (between 20% and 30%) under different phantom images and values of *c* maintained greater than 0.9. It indicated that this algorithm was more stable and suitable for a wide range of situations.

(3)  The criteria values of the reconstructed particle phantom were better than those of the head phantom. This may be caused by the different complexity of the phantom images. Though the particle phantom involved large number of particle elements, they were generally in the same size. And there were only two gray levels in the image. So the complexity of the particle phantom could be considered to be relatively low. On the contrary, in the head phantom, the size of the ellipse varies from small to large and there were six gray levels in this phantom. So the head phantom is more complex than the particle phantom and it is more difficult to reconstruct it.

### 3.3. Local Reconstruction Using Real Experimental Data

**Figure 8:**
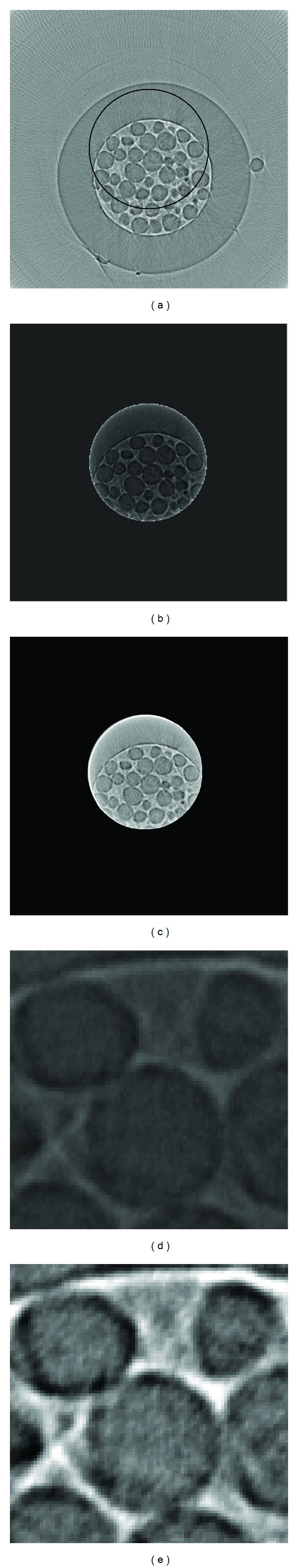
(a) Reconstruction by FBP using global data. (b) Reconstruction by FBP using local data. (c) Reconstruction by the new algorithm using local data. (d) and (e) were the amplifications of (b) and (c), respectively.

In previous section, the effectiveness of this new wavelet method was validated by two phantom images. In this section the method was applied to practical data collected in a CT experiment.  Figure 8(a) showed a slice of the sample reconstructed by FBP. The projections had been collected at 180 angles over 180 degrees. And the length of each projection was 1207 pixels. Using the new wavelet algorithm, a local centered region of radius 150 pixels had been reconstructed. So only 25% of the projections were used in the reconstruction (see Figure 8(c)). The reconstructed local region was as good as what could be obtained using the FBP and global data. For comparison, the local region was reconstructed using the FBP and local data and the result was shown in Figure 8(b). The amplifications of the images reconstructed by the new method and FBP were, respectively, shown in Figures 8(d) and 8(e). It could be seen that the image was brighter and the boundaries of particles are clearer in Figure 8(e). This fact indicated that the wavelet method had advantages over the FBP when reconstructing local region.

## 4. Conclusion

Based on the properties of wavelet, a local reconstruction algorithm has been proposed and implemented. It has been observed that for some wavelet bases with many vanishing moments, the scaling and wavelet functions have essentially the same support after differentiation and Hilbert transform. According to this fact, a local reconstruction scheme has been developed to reconstruct a local region of a cross-section of a sample with essentially local data. Some experiments have been performed and the results confirmed the effectiveness of this new algorithm. Because this algorithm is able to reconstruct local small region in a large sample that exceeds the view field, it may contribute to make more kinds of materials appropriate for CT investigation whose spatial resolution can achieve micro-/nanoscale. However, there are still some unsolved problems. For example, when using different wavelet functions, the results of the algorithm were different. But how to select a suitable wavelet function for a certain situation is still no solution.

## Figures and Tables

**Table 1:** Criteria values for the local reconstruction of the stacked particles phantom.

Algorithm	*d*	Relative change of *d*	*r*	Relative change of *r*	*c*	Relative change of *c*
FBP	0.6446	—	0.7782	—	0.7896	—
Method in [13]	0.4347	↑32.56%	0.5295	↑31.96%	0.9047	↑14.58%
Method in this paper	0.3918	↑39.22%	0.4749	↑38.97%	0.9222	↑16.79%

**Table 2:** Criteria values for the local reconstruction of the Shepp-Logan head phantom.

Algorithm	*d*	Relative change of *d*	*r*	Relative change of *r*	*c*	Relative change of *c*
FBP	1.8632	—	2.6380	—	0.7095	—
Method in [13]	1.7999	↑3.40%	2.5667	↑2.70%	0.8116	↑14.4%
Method in this paper	1.5042	↑19.27%	1.8156	↑31.18%	0.9796	↑38.07%
